# Characterization of *bla*_KPC-2_-Carrying Plasmid pR31-KPC from a *Pseudomonas aeruginosa* Strain Isolated in China

**DOI:** 10.3390/antibiotics10101234

**Published:** 2021-10-11

**Authors:** Min Yuan, Hongxia Guan, Dan Sha, Wenting Cao, Xiaofeng Song, Jie Che, Biao Kan, Juan Li

**Affiliations:** 1 State Key Laboratory for Infectious Diseases Prevention and Control, Collaborative Innovation Center for Diagnosis and Treatment of Infectious Disease, National Institute for Communicable Disease Control and Prevention, Chinese Center for Disease Control and Prevention, Beijing 102206, China; yuanmin@icdc.cn (M.Y.); songxiaofeng@icdc.cn (X.S.); chejie@icdc.cn (J.C.); kanbiao@icdc.cn (B.K.); 2 Wuxi Center for Disease Control and Prevention, Wuxi 214023, China; ghx-331@163.com (H.G.); shadan20051001@163.com (D.S.); caowent1978@126.com (W.C.)

**Keywords:** *Pseudomonas aeruginosa*, carbapenem resistance, KPC-2, plasmid

## Abstract

This work aimed to characterize a 29-kb *bla*_KPC-2_-carrying plasmid, pR31-KPC, from a multidrug resistant strain of *Pseudomonas aeruginosa* isolated from the sputum of an elderly patient with multiple chronic conditions in China. The backbone of pR31-KPC is closely related to four other *bla*_KPC-2_-carrying plasmids, YLH6_p3, p1011-KPC2, p14057A, and pP23-KPC, none of which have been assigned to any of the known incompatibility groups. Two accessory modules, the IS*26*-*bla*_KPC-2_-IS*26* unit and IS*26*-ΔTn*6376*-IS*26* region, separated by a 5.9-kb backbone region, were identified in pR31-KPC, which was also shown to carry the unique resistance marker *bla*_KPC-2_. A comparative study of the above five plasmids showed that p1011-KPC2 may be the most complete plasmid of this group to be reported, while pR31-KPC is the smallest plasmid having lost most of its conjugative region. Regions between the iterons and *orf207* in the backbone may be hot spots for the acquisition of exogenous resistance entities. The accessory regions of these plasmids have all undergone several biological events when compared with Tn*6296*. The further transfer of *bla*_KPC-2_ in these plasmids may be initiated by either the Tn*3* family or IS*26*-associated transposition or homologous recombination. The data presented here will contribute to a deeper understanding of *bla*_KPC-2_ carrying plasmids in *Pseudomonas*.

## 1. Introduction

*Pseudomonas aeruginosa* is ubiquitous and is a well-known opportunistic pathogen in hospitalized, immunocompromised patients. *P. aeruginosa* is capable of infecting all types of systems and tissues, causing various diseases, such as bacteremia, septicemia, septicopyemia, pneumonia, bronchitis, diarrhea, keratitis, and skin and wound infections [1,2]. Multidrug resistant (MDR) *P. aeruginosa* has been categorized as a serious health threat by the Centers for Disease Control and Prevention since 2019 (https://www.cdc.gov/drugresistance/biggest_threats.html), and the development of resistance to carbapenem will further exacerbate the situation [3].

Resistance to carbapenemase in *P. aeruginosa* is generally due to a combination of mechanisms, including porin (OprD) deficiency, overexpression of efflux pumps, intrinsic chromosomally encoded AmpC-lactamase, and/or carbapenemase production [4]. Carbapanemase-encoding genes are highly transferable as they always reside on mobile genetic elements (MGEs) such as plasmids, gene cassettes of integrons, transposons, and genomic islands [5]. To date, the MGE-related carbapenemase families that have been reported in *P. aeruginosa* include class A (KPC-2 [6] and GES [7]), class B (IMP [8], VIM [9], SPM [10], NDM [11], AIM [12], SIM [13], FIM [14], HMB [15], and CAM [16]), and class D (OXA-40-like [17], OXA-48-like [18], and OXA-198-like [19]). *P. aeruginosa* is the predominant host for class B enzymes, while class A and D enzymes are less commonly reported in this species [20].

The KPC-2-producing *P. aeruginosa* isolates were first reported in 2006 in a strain from Colombia [6] and have recently been reported in strains from North America [21], South America (Trinidad and Tobago [22], South Florida [23], and Puerto Rico [24]), China [25], Brazil [26], and Germany [27]. The *bla*_KPC-2_ gene can be either plasmid-borne or on the chromosome of the host. Using the keywords “pseudomonas”, “KPC”, “plasmid”, and “complete” to search the GenBank database (last accessed on 14 May 2021), a total of six fully sequenced and published *bla*_KPC-2_-carrying plasmids from *P. aeruginosa* were obtained: Two IncP-6 plasmids, pCOL-1 (accession number KC609323; from Colombia) [28] and p10265-KPC (accession number KU578314; from China) [29]; one IncU plasmid, pPA-2 (accession number KC609322; from Colombia) [28]; plasmid pBH6 (accession number CP029714; from Brazil) [30]; and p1011-KPC2 and p14057A (accession numbers MH734334, KY296095, and CP065418; all from China) [31,32], which all belong to the same unknown incompatibility group.

In this work, we characterized the *bla*_KPC-2_-carrying *P. aeruginosa* plasmid pR31-KPC and compared its sequence with those of the sequenced plasmids in the database to gain a deeper understanding of the evolutionary history of this group of plasmids. Tn*6774* was the novel transposon designated in this study.

## 2. Results and Discussion

### 2.1. Case Report 

On 31 August 2015, a 79-year-old man was admitted to a local hospital in Yixing City with a cough and expectoration that had persisted for half a year, which showed progressive aggravation for 20 h. The patient was subsequently diagnosed with pneumonia, hypertension, sequela of cerebral apoplexy, and post tracheotomy. The patient received a series of symptomatic treatments, such as aspiration of sputum to relieve the cough, oxygen inhalation to improve circulation, and intravenous administration of meropenem and tigecycline to reduce inflammation. *P. aeruginosa* R31 was isolated from a sputum specimen during hospitalization. On the eighteenth day of hospitalization, his symptoms worsened and he displayed shortness of breath, decreased blood pressure, decreased urine output, and symptoms of multiple organ dysfunction.

### 2.2. General Features of P. aeruginosa R31

Strain R31 was highly resistant to all of the β-lactams tested, including penicillins (piperacillin, piperacillin/tazobactam), cephalosporins (ceftazidime, cefepime), carbapenems (imipenem, meropenem), and monobactam (aztreonam). Moreover, it showed resistance to some fluoroquinolones (ciprofloxacin, levofloxacin), but was still susceptible to aminoglycosides (gentamicin, amikacin, and tobramycin) and colistin (Table 1).

The R31 strain returned a positive result in the Carba NP test, and out of all the carbapenemase genes tested, only *bla*_KPC-2_ was detected. Repeated conjugation experiments failed to transfer the *bla*_KPC-2_ marker from R31 to *P. aeruginosa* PAO1 (induced rifampin resistance) or Escherichia coli EC600 (rifampin resistance).

### 2.3. Overview of pR31-KPC

The R31 isolate harbors only one extrachromosomal closed circular DNA sequence, designated as pR31-KPC, which was determined to be 29,402 bp in length and contain a mean G+C content of 57.5% and 44 predicted open reading frames (ORFs) (Figure S1). The backbone of pR31-KPC has a modular structure, with the insertion of two accessory modules: The IS26-*bla*_KPC-2_-IS26 unit and IS26-∆Tn6376-IS26 region. The accessory modules were defined as acquired DNA regions associated with and bordered by mobile elements.

### 2.4. The Backbone of pR31-KPC

The backbone of pR31-KPC is 16.9-kb in length and contains the following elements: The RepA and its iterons (repeat region for the RepA binding site), which are responsible for plasmid replication initiation. The iterons are 137 bp in size, within which 12-bp sites are located, relatively conserved, and repeated six times; parA for plasmid partition; higBA, which encodes the toxin-antitoxin system for post-segregational killing; and a traMLI conjugation system remnant. The identified RepA protein of pR31-KPC showed a 100% amino acid similarity to the homologs in the four other *bla*_KPC-2_-carrying Pseudomonas plasmids of the same incompatibility group, which are available in public sequence databases, namely p1011-KPC2, p14057A, YLH6_P3 (accession number MK882885), and pP23-KPC (accession number CP065418).

**Figure 1:**
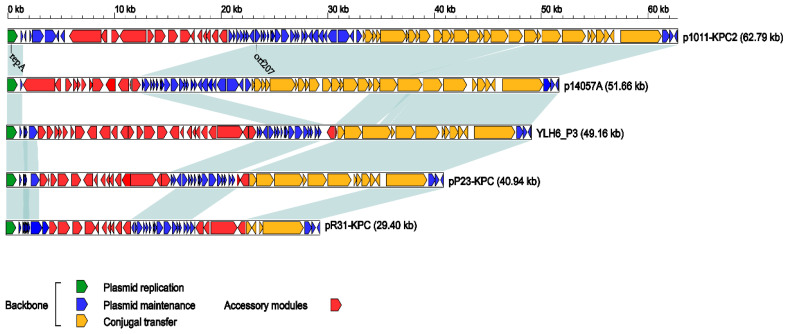
Linear comparison of plasmid genome sequences. Genes are denoted by arrows. The plasmid backbone replication, maintenance, and conjugation regions are colored in green, dark blue, and orange, respectively. The accessory module regions are colored in red. Shading denotes homology (nucleotide identity ≥ 90%) of the plasmid backbone regions, but not the accessory modules. *RepA* and *orf207* represent the names of the labeled genes, respectively. The GenBank accession numbers of p1011-KPC2, p14057A, YLH6_P3, pP23-KPC, and pR31-KPC are MH734334, KY296095, MK882885, CP065418, and CP061851, respectively.

The backbone of pR31-KPC showed 83–100% coverage and 100% identity to the above-mentioned plasmids (Supplementary Table S1). A linear comparison of the backbones of these five plasmids revealed the following: (1) The regions between the iterons and orf207 are hot spots for the acquisition of resistance genes, and all of the *bla*_KPC-2_ genes reside in these regions; (2) p1011-KPC2 is the most complete plasmid of this incompatibility group, with a complete conjugative region and a relatively intact maintenance region, while pR31-KPC is the smallest plasmid of this group (Figure 1). Although most of its conjugative region is missing and is unable to conjugate experimentally, pR31-KPC and thus, *bla*_KPC-2_ can remain in its host.

### 2.5. The Accessory Regions of pR31-KPC

**Figure 2:**
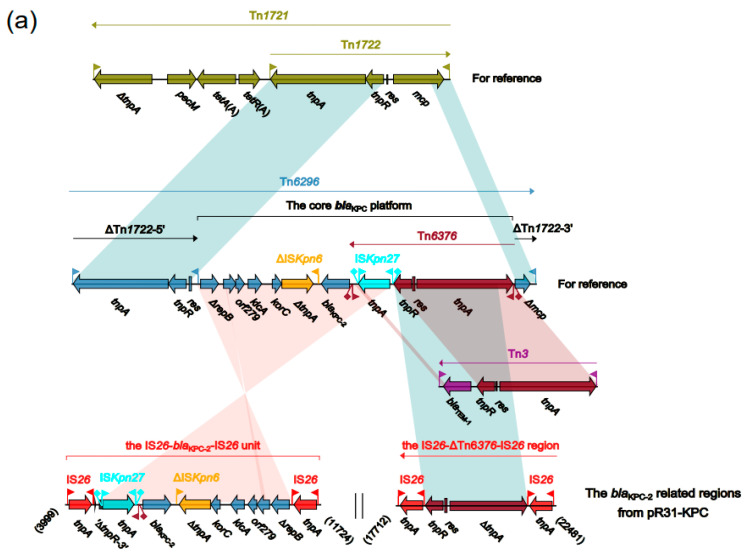

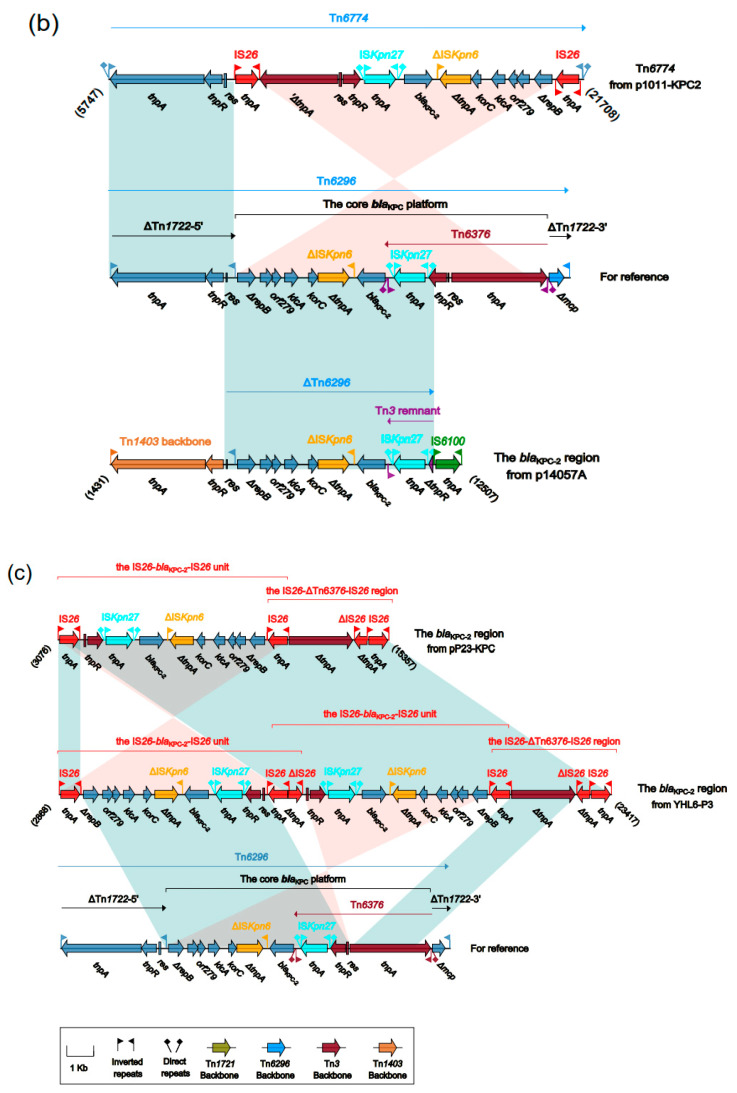
(**a**) The accessory regions pR31-KPC, and comparison with Tn*6296* and Tn*1721*; (**b**) The accessory regions p1011-KPC2 and p14057A, and comparison with Tn*6296*; (**c**) The accessory regions of pP23-KPC and YLH6_P3, and comparison with Tn*6296*. Genes are denoted by arrows. Genes, mobile elements, and other features are colored based on function classification. Shading denotes regions of homology (>95% nucleotide identity). Numbers in brackets indicate the nucleotide positions within the corresponding plasmids. The GenBank accession numbers of Tn*1721*, Tn*3*, Tn*6296*, and Tn*1403* are X61367, HM749966, FJ628167, and AF313472, respectively.

The accessory regions of pR31-KPC comprise two IS26-based regions, the IS26-*bla*_KPC-2_-IS26 unit and IS26-∆Tn6376-IS26 region, separated by a backbone region of 5988 bp (Figure 2a).

Tn6296 is widely considered to be one of the most important vehicles for *bla*_KPC-2_ gene transferring. Tn6296 was originally identified in MDR plasmid pKP048 from *Klebsiella pneumoniae*. In addition, it was generated from the insertion of the core *bla*_KPC-2_ genetic platform (Tn6376–*bla*_KPC-2_–ΔISKpn6–korC–orf6–klcA–ΔrepB) into Tn1722, resulting in truncation of mcp (Figure 2a).

In pR31-KPC, the aforementioned core *bla*_KPC-2_ genetic platform is intact, but has been split into two parts, each of which is bordered by two IS26 elements (either in the same or opposite directions), generating the IS26-*bla*_KPC-2_-IS26 unit and IS26-∆Tn6376-IS26 region, which have the potential to move (Figure 2a). Both of the regions lack the typical 5 bp target site duplications, suggesting that the acquisition of these entities may have occurred via the IS26-mediated homologous recombination.

In p1011-KPC2, two copies of IS26 were found at the boundaries of the core *bla*_KPC-2_ genetic platform in opposite directions, translocating the core platform and truncating tnpA_Tn6376_ into a 2455 bp fragment. Regarding the integrity of Tn6296, the left/right inverted repeats and direct repeats were not impaired, generating the novel transposon Tn6774 (Figure 2b). The further spread of *bla*_KPC-2_ may occur by either the Tn6774 transposition via a TnpA/TnpR_Tn6774_-mediated ‘cut and paste’ process or IS26-mediated transposition.

In p14057A, Tn6296 was truncated by the Tn1403 core tni module and IS6100 at either ends, generating the ΔTn1403-ΔTn6296-IS6100 region (Figure 2b). This entity may have been generated by a recombination of Tn6296 and a Tn1403-like transposon at the res site. Tn1403, initially found in Pseudomonas, is an important resistance gene dissemination vehicle, with the derivatives Tn6060, Tn6061, Tn6217, Tn6249, and Tn6286 having been reported in [33,34,35,36,37]. Belonging to the Tn21 subfamily of the Tn3 family, the Tn1403 and Tn1403-like transposons are able to transfer their passengers by the one-end transposition [38].

In YLH6_P3 and pP23-KPC, six copies of IS26 (four intact and two truncated) and four copies of IS26 (three intact and one truncated) were found in the *bla*_KPC-2_ region, respectively, forming mosaic structures, with adjacent IS26 regions overlapping each other. In YLH6_P3, two copies of *bla*_KPC-2_ were found. This structure was likely generated by the duplication of IS26-*bla*_KPC-2_-IS26 found in pP23-KPC or vice versa. Linkage to IS26 indicates the potential for further dissemination of *bla*_KPC-2_ (Figure 2c).

### 2.6. Genomic Characterization of P. aeruginosa Genomes 

A total of 209 genomes were downloaded (including that of R31) from the GenBank database. The resistance genes carried by each genome are listed in Table S2. All of the genomes, except for seven, have the chromosome-origin *bla*_PAO-1_ gene. The seven genomes without the gene were excluded for further whole genome phylogeny studies, due to the probable misidentification at the genus or species level. Phylogeny studies of the remaining 202 genomes revealed the following: (1) These clones can be divided into three clusters (clusters I, II, and III), and cluster III can be further divided into IIIa and IIIb. R31 belongs to cluster IIIa. (2) The carriage rates of carbapenemase genes for clusters I, II, IIIa, and IIIb were 33.3% (3/9), 36.45% (35/96), 23% (8/26), and 2.8% (2/71), respectively. Compared with clusters I, II, and IIIa, cluster IIIb clones have the lowest carriage rate of carbapenemase genes. (3) Worldwide, the sequence types (STs) of sequenced *P. aeruginosa* genomes are highly diverse.

The 202 genomes included a total of 69 known STs and 36 unknown STs. Forty-eight isolates with 23 different kinds of carbapenemase genotypes included 17 known STs and nine unknowns STs. A specific relationship between the STs and carbapenemase genotypes was not obvious (Figure S2).

## 3. Materials and Methods

### 3.1. Ethics Statement

The specimens were acquired with consent from the patient. The use of human specimens and all of the related experimental protocols was reviewed and approved by the ethics committee of the National Institute for Communicable Disease Control and Prevention (ICDC), Beijing, China, in accordance with the medical research regulations of the Ministry of Health, China. Research involving biohazardous materials and all of the related procedures were approved by the Biosafety Committee of the ICDC. This study was conducted in China.

### 3.2. Identification of Bacterial Strains 

Bacterial species were identified with the VITEK-2 Compact system using the GNI card (bioMerieux, France) and further confirmed by sequencing of the 16S rDNA amplicon, which is generated by primer pairs 27f (5′-AGAGTTTGATCCTGGCTCAG-3′) and 1492r (5′-ACGGCTACCTTGTTACGACTT-3′).

### 3.3. Determination of Minimum Inhibitory Concentration (MIC)

Antimicrobial susceptibility testing was performed by a broth microdilution method with customized microtiter plates containing vacuum dried antibiotics (BD Bioscience, San Jose, CA, USA). The MIC values were interpreted according to the Clinical and Laboratory Standards Institute (CLSI) guidelines 2019.

### 3.4. Detection of Carbapenemase Activity and Screening of Responsible Genes

The Carba NP test recommended by CLSI was performed for the detection of the carbapenemase production. The major plasmid-borne carbapenemase genes were amplified by the polymerase chain reaction (PCR) and sequenced on an ABI 3730 DNA Analyzer (Applied Biosystems, Foster City, CA, USA).

### 3.5. Conjugation Experiments

Conjugation experiments were carried out by a filter mating method with *P. aeruginosa* R31 as the donor strain and *P. aeruginosa* PAO1 (induced rifampin resistance) or *E. coli* EC600 (rifampin resistance) serving as the recipient strain. Briefly, the donor and recipient strains were grown in 3 mL of brain heart infusion (BHI) broth overnight at 37 °C. For each conjugation, 50 μL of donor strain culture was mixed with 500 μL of recipient strain culture (v:v = 1:10) and 4.5 mL of fresh BHI broth. In addition, 100 μL of the mixture was applied onto a cellulose filter membrane (pore size, 0.22 μm) already placed on a BHI agar plate. After incubation at 37 °C for 16–18 h, the filter membrane was taken out and vortexed in 1 mL of BHI broth. The vortex mixtures were plated on BHI agar plates containing 100 mg/L ceftazidime and 50 mg/L rifampin for the selection of the *P. aeruginosa* PAO1 transconjugants or on BHI agar plates containing 100 mg/L ceftazidime and 100 mg/L sodium azide for the selection of the *E. coli* EC600 transconjugants.

### 3.6. Determination of R31 Genome Sequences

Genomic DNA was isolated from the R31 isolate using a Wizard Genomic DNA Purification Kit (Promega, Madison, WI, USA), and sequenced using a whole-genome shotgun strategy on an Ion Torrent Personal Genome Machine system (Life Technologies). A Pacific Biosciences RSII DNA sequencing system (Menlo Park, CA, USA) was used as the platform to sequence the complete genome. The contigs were assembled using Hgap 2.0.

### 3.7. Sequence Annotation and Comparison

Sequence annotation and prediction of open reading frames (ORF) and pseudogenes of plasmid pR31-KPC were performed using RAST 2.0 [39] and further annotated by BLASTP/BLASTN [40] searches against the UniProtKB/Swiss-Prot [41] and RefSeq [42] databases. Annotation of resistance genes, mobile elements, and other features was conducted using online databases, such as CARD [43], ResFinder [44], ISfinder [45], ISsaga [46], INTEGRALL [47], and the Tn Number Registry [48]. Multiple and pairwise sequence comparisons were performed using MUSCLE 3.8.31 [49] and BLASTN, respectively. Gene organization diagrams were drawn in Inkscape 0.48.1 (http://inkscape.org, accessed on 1 January 2019)

### 3.8. Whole Genome Phylogeny and Genetic Background Analysis 

The available whole genome sequences of *P. aeruginosa* from the National Center for Biotechnology Information (NCBI) were downloaded (last accessed on 24 October 2019). Resistance genes of the Pseudomonas genomes were analyzed by ResFinder. The sequence types (ST) of these *P. aeruginosa* genomes were obtained using the webtool PubMLST (https://pubmlst.org/organisms/pseudomonas-aeruginosa). Analyses of the downloaded genomes (including R31) were further processed using kSNP3 v3.1 [50]. Core single-nucleotide polymorphism (SNP) matrices were generated, and maximum-likelihood phylogenies were constructed. Phylogenetic trees were drawn using the Interactive Tree of Life (iTOL) programs [51].

### 3.9. Nucleotide Sequence Accession Number

The complete sequences of the chromosome of R31 and plasmid pR31-KPC were submitted to GenBank database, under accession numbers CP061850 and CP061851, respectively.

## 4. Conclusions

Carbapenems are still first-line agents for the treatment of *P. aeruginosa* infections. The occurrence and dissemination of *bla*_KPC-2_ and/or other carbapenemase gene-carrying *P. aeruginosa* strains have important clinical and epidemiological implications. Four other fully sequenced plasmids of the same incompatibility group as pR31-KPC have been reported in China, which suggest that it may be an important vehicle in the dissemination of *bla*_KPC-2_ in *Pseudomonas*.

## Figures and Tables

**Table 1:** Minimum inhibitory concentration (MIC) values of *P. aeruginosa* R31 determined by the microdilution method.

	MIC Values			MIC Breakpoints µg/mL	
	µg/mL	R or S	S	I	R
Piperacillin	>1024	R	≤16	32–64	≥128
Piperacillin/tazobactam	>1024/4	R	≤16/4	32/4–64/4	≥128/4
Ceftazidime	128	R	≤8	16	≥32
Cefepime	>512	R	≤8	16	≥32
Imipenem	>128	R	≤2	4	≥8
Meropenem	>128	R	≤2	4	≥8
Aztreonam	>512	R	≤8	16	≥32
Gentamicin	4	S	≤4	8	≥16
Amikacin	<8	S	≤16	32	≥64
Tobramycin	<2	S	≤4	8	≥16
Ciprofloxacin	8	R	≤0.5	1	≥2
Levofloxacin	32	R	≤1	2	≥4
Colistin	1	S	≤2	-	≥4

## Data Availability

The data presented in this study are available on request from the corresponding author. The plasmid sequences analyzed in this study can be found in public NCBI Genbank databases. The accession numbers were provided in this article when these plasmids were firstly indicated.

## Supplementary Materials

The following are available online at https://www.mdpi.com/article/10.3390/antibiotics10101234/s1. Figure S1: Schematic maps of p1011-KPC2, p14057A, YLH6_P3, pP23-KPC, and pR31-KPC. Figure S2: Evolutionary relationships of 202 *P. aeruginosa* genomes in the GenBank database. Table S1: Pairwise comparison of *bla*_KPC-2_-carrying plasmids from *P. aeruginosa* using BLASTN. Table S2: Beta-lactamase and carbapenemase genes carried by *P. aeruginosa* genomes based on ResFinder results.
