# Supplementary material for: Characterization of blaKPC-2-Carrying Plasmid pR31-KPC from a Pseudomonas aeruginosa Strain Isolated in China
Source: Antibiotics (Basel). 2021 Oct 11;10(10):1234. doi: 10.3390/antibiotics10101234 (PMC8532800; doi:10.3390/antibiotics10101234)
Supplement: Supplementary file 1 [file antibiotics-10-01234-s001.zip › antibiotics-1357091-supplementary.pdf]

Supplementary Material

Figure S1. Schematic maps of p1011-KPC2, p14057A, YLH6_P3, pP23-KPC, and pR31-KPC. Genes are denoted by arrows, and the backbone and accessory module regions are in different colors. The innermost circle presents GC-skew [(G-C)/(G+C)], with a window size of 500 bp and a step size of 20 bp. The next-to-innermost circle presents GC content. The accession numbers of p1011-KPC2, p14057A, YLH6_P3 and pP23-KPC for reference are MH734334, KY296095, MK882885, CP065418, respectively.

Figure S2. The maximum parsimony phylogenetic tree of the 202 *P. aeruginosa* genomes in the GenBank database based on SNP. The strain names, sequence types, the name of carbapenemase genes carried by *P. aeruginosa* were labeled. The column in grey, light green, dark green and red indicate the numbers of carbapeneamse genes carried by each isolate were 0,1,2 and 3 respectively.

Table S1. Pairwise comparison of $\textit{bla}_{\text{KPC-2}}$-carrying plasmids from *P. aeruginosa* using BlastN.

Table S2. Beta-lactamase and carbapenmase genes carried by *P. aeruginosa* genomes

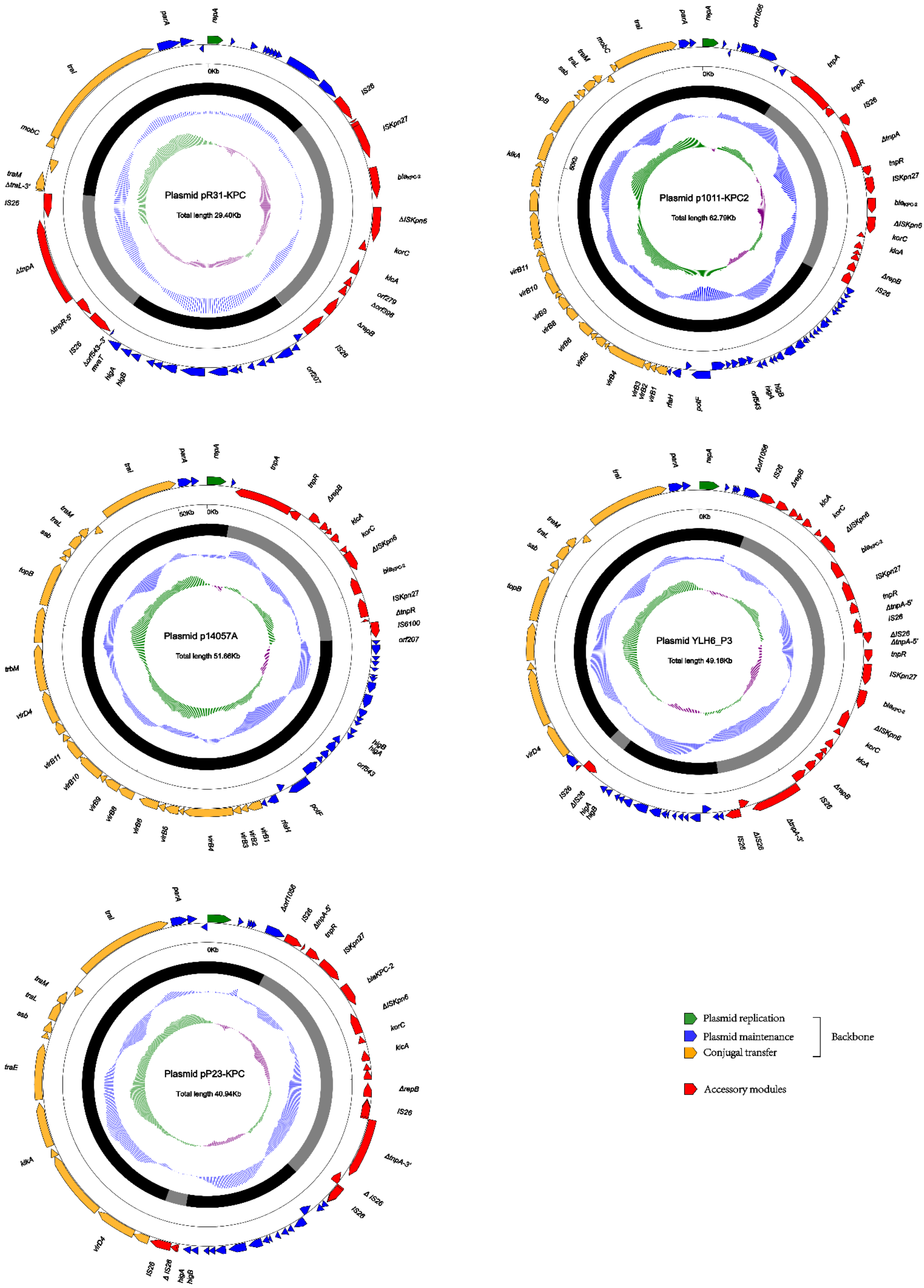

Plasmid pR31-KPC
Total length 29.40Kb
0Kb
repA
parA
traI
mobC
traM
ΔtraL-3'
IS26
ΔtnpA
ΔtnpR-5'
IS26
Δorf543-3'
mvaT
higA
higB
orf207
IS26
ΔrepB
Δorf396
orf279
klcA
korC
ΔISKpn6
blaKPC-2
ISKpn27
IS26
Plasmid p1011-KPC2
Total length 62.79Kb
0Kb
60Kb
repA
parA
traI
mobC
traM
traL
ssb
topB
klkA
virB11
virB10
virB9
virB8
virB6
virB5
virB4
virB3
virB2
virB1
finH
pcdF
orf543
higA
higB
IS26
ΔrepB
klcA
korC
ΔISKpn6
blaKPC-2
ISKpn27
tnpR
ΔtnpA
IS26
tnpR
tnpA
orf1056
Plasmid p14057A
Total length 51.66Kb
50Kb
0Kb
repA
parA
traI
traM
traL
ssb
topB
trbM
virD4
virB11
virB10
virB9
virB8
virB6
virB5
virB4
virB3
virB2
virB1
finH
pcdF
orf543
higA
higB
orf207
IS6100
ΔtnpR
ISKpn27
blaKPC-2
ΔISKpn6
korC
klcA
ΔrepB
tnpR
tnpA
Plasmid YLH6_P3
Total length 49.16Kb
0Kb
repA
parA
traI
traM
traL
ssb
topB
virD4
IS26
ΔIS26
higA
higB
IS26
ΔIS26
ΔtnpA-3'
IS26
ΔrepB
klcA
korC
ΔISKpn6
blaKPC-2
ISKpn27
tnpR
ΔtnpA-5'
ΔIS26
IS26
ΔtnpA-5'
tnpR
ISKpn27
blaKPC-2
ΔISKpn6
korC
klcA
ΔrepB
IS26
Δorf1056
Plasmid pP23-KPC
Total length 40.94Kb
0Kb
parA
traI
traM
traL
ssb
traE
klkA
virD4
IS26
Δ IS26
higA
higB
IS26
Δ IS26
ΔtnpA-3'
IS26
ΔrepB
klcA
korC
ΔISKpn6
blaKPC-2
ISKpn27
tnpR
ΔtnpA-5'
IS26
Δorf1056
Plasmid replication
Plasmid maintenance
Conjugal transfer
Backbone
Accessory modules

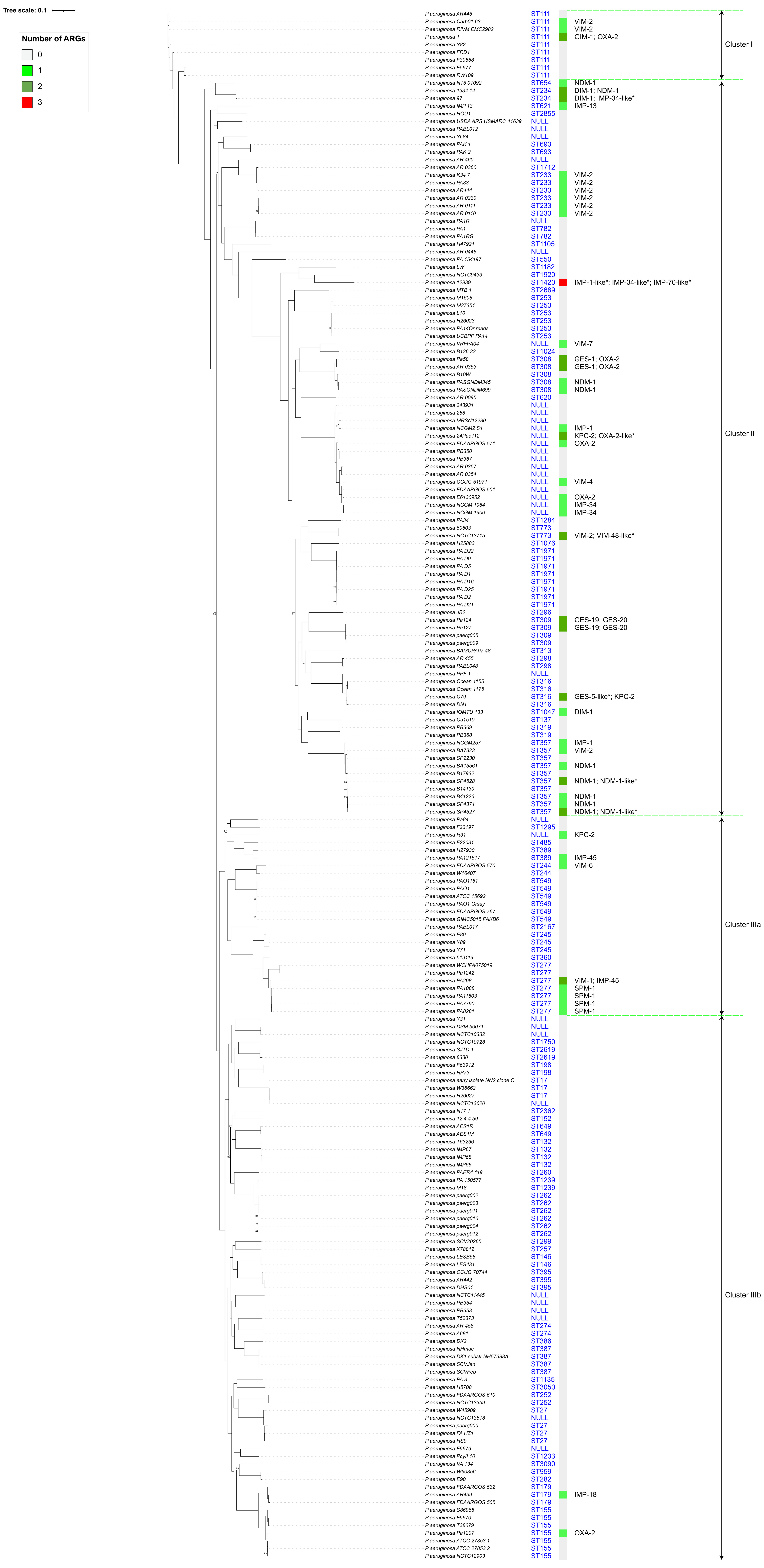

Tree scale: 0.1
Number of ARGs
0
1
2
3
VIM-2
VIM-2
GIM-1; OXA-2
Cluster I
NDM-1
DIM-1; NDM-1
DIM-1; IMP-34-like*
IMP-13
VIM-2
VIM-2
VIM-2
VIM-2
VIM-2
VIM-2
IMP-1-like*; IMP-34-like*; IMP-70-like*
VIM-7
GES-1; OXA-2
GES-1; OXA-2
NDM-1
NDM-1
IMP-1
KPC-2; OXA-2-like*
OXA-2
VIM-4
OXA-2
IMP-34
IMP-34
VIM-2; VIM-48-like*
GES-19; GES-20
GES-19; GES-20
GES-5-like*; KPC-2
DIM-1
IMP-1
VIM-2
NDM-1
NDM-1; NDM-1-like*
NDM-1
NDM-1
NDM-1; NDM-1-like*
Cluster II
KPC-2
IMP-45
VIM-6
VIM-1; IMP-45
SPM-1
SPM-1
SPM-1
SPM-1
Cluster IIIa
IMP-18
OXA-2
Cluster IIIb

Table S1. Pairwise comparison of *bla*$_{KPC-2}$-carrying plasmids from *Pseudomonas aeruginosa* using BlastN

(coverage+identity)	YLH6_P3	p1011-KPC2	p14057-KPC	pP23-KPC	pR31-KPC
YLH6_P3		(100%+99.22%)	(85%+98.78%)	(99%+100%)	(75%+100%)
p1011-KPC2	(61%+99.22%)		(84%+99.79%)	(61%+99.22%)	(44%+100%)
p14057-KPC	(60%+98.78%)	(99%+99.79%)		(60%+99.78%)	(40%+100%)
pP23-KPC	(100%+100%)	(100%+99.22%)	(84%+98.78%)		(71%+100%)
pR31-KPC	(95%+100%)	(100%+100%)	(78%+100%)	(95%+100%)	

**Table S2 Beta-lactamase and carbapenmase genes carried by *P.aeruginosa* genomes based on Resfinder results**				
**Strain name**	**Genebank accession No.**	**Genome component**	**Beta-lactamase (coverage/identity)**	**Carbapenemase(coverage/identity)**
P.aeruginosa 1	NZ_LS998783<br>NZ_LS998784	Chromosome<br>plasmid (unnamed)	*bla*$_{OXA-395}$ (100/100)<br>*bla*$_{PAO}$ (100/99.49)	*bla*$_{GIM-1}$ (100/100)<br>*bla*$_{OXA-2}$ (100/100)
P.aeruginosa 24Pae112	NZ_CP029605	Chromosome	bla$_{OXA-488}$ (100/99.87)<br>*bla*$_{PAO}$ (100/99.24)	*bla*$_{KPC-2}$ (100/100)<br>bla$_{OXA-2/15}$ (100/99.87)
P.aeruginosa 97	NZ_CP031449	Chromosome	*bla*$_{OXA-395}$ (100/99.24)<br>*bla*$_{PAO}$ (100/98.99)<br>*bla*$_{OXA-10}$ (100/100)<br>*bla*$_{OXA-129}$ (100/100)	*bla*$_{IMP-34}$ (100/99.73)<br>*bla*$_{DIM-1}$ (100/100)
P.aeruginosa 1334/14	NZ_CP035739	Chromosome	*bla*$_{OXA-486}$ (100/99.74)<br>*bla*$_{PAO}$ (100/99.83)<br>*bla*$_{PME-1}$ (100/100)<br>*bla*$_{OXA-10}$ (100/100)	*bla*$_{DIM-1}$ (100/100)<br>*bla*$_{NDM-1}$ (100/100)
P.aeruginosa 12939	NZ_CP024477	Chromosome	*bla*$_{OXA-395}$ (100/99.87)<br>*bla*$_{PAO}$ (100/99.24)	*bla*$_{IMP-1/34/70}$(100/99.46)
P.aeruginosa AR_0110	NZ_CP029745	Chromosome	*bla*$_{OXA-486}$ (100/99.49)<br>*bla*$_{PAO}$ (100/99.74)	*bla*$_{VIM-2}$ (100/100)
P.aeruginosa AR_0111	NZ_CP032257<br>NZ_CP032256	Chromosome<br>plasmid (unnamed)	*bla*$_{OXA-486}$ (100/99.74)<br>*bla*$_{PAO}$ (100/99.49)<br>*bla*$_{OXA-4}$ (100/100)	*bla*$_{VIM-2}$ (100/100)

P.aeruginosa AR_0230	NZ_CP027174<br>NZ_CP027175<br>NZ_CP027176	Chromosome<br><br>plasmid(unnamed)<br><br>plasmid(unnamed)	*bla*$_{OXA-486}$ (100/99.74)<br>*bla*$_{PAO}$ (100/99.49)<br>*bla*$_{OXA-4}$ (100/100)	*bla*$_{VIM-2}$ (100/100)
P.aeruginosa AR_0353	NZ_CP027172<br>NZ_CP027173	Chromosome<br>plasmid(unnamed)	*bla*$_{OXA-486}$ (100/100)<br>*bla*$_{PAO}$ (100/99.24)	*bla*$_{GES-1}$ (100/100)<br>*bla*$_{OXA-2}$ (100/100)
P.aeruginosa AR439	NZ_CP029097<br>NZ_CP029095<br>NZ_CP029096	Chromosome<br>plasmid(unnamed)<br>plasmid(unnamed)	*bla*$_{OXA-396}$ (100/100)<br>*bla*$_{PAO}$ (100/99.58)	*bla*$_{IMP-18}$ (100/100)
P.aeruginosa AR444	NZ_CP029089	Chromosome	*bla*$_{OXA-486}$ (100/99.74)<br>*bla*$_{PAO}$ (100/99.49)<br>*bla*$_{OXA-4}$ (100/100)	*bla*$_{VIM-2}$ (100/100)
P.aeruginosa B41226	NZ_CP034368	Chromosome	*bla*$_{OXA-50}$ (100/99.36)<br>*bla*$_{PAO}$ (100/99.49)<br>*bla*$_{VEB-1}$ (100/100)<br>*bla*$_{OXA-10}$ (100/99.87)	*bla*$_{NDM-1}$ (100/100)
P.aeruginosa BA7823	NZ_CP032569	Chromosome	*bla*$_{OXA-50}$ (100/99.36)<br>*bla*$_{PAO}$ (100/99.49)<br>*bla*$_{LCR-1}$ (100/100)	*bla*$_{VIM-2}$ (100/100)

P.aeruginosa BA15561	NZ_CP033432	Chromosome	*bla*$_{OXA-50}$ (100/99.36)<br>*bla*$_{PAO}$ (100/99.49)<br>*bla*$_{VEB-1}$ (100/100)<br>*bla*$_{OXA-10/246}$ (100/99.62)	*bla*$_{NDM-1}$ (100/100)
P.aeruginosa C79	NZ_CP040684<br>NZ_CP040685	Chromosome<br>plasmid (p1)	*bla*$_{OXA-395}$ (100/100)<br>*bla*$_{PAO}$ (100/98.99)	*bla*$_{GES-5}$ (100/100)<br>*bla*$_{KPC-2}$ (100/100)
P.aeruginosa Carb01 63	NZ_CP011317	Chromosome	bla$_{OXA-395}$ (100/100)bla$_{PAO}$ (100/99.49)bla$_{CARB-2}$ (100/100)	*bla*$_{VIM-2}$ (100/100)
P.aeruginosa CCUG 51971	NZ_CP043328	Chromosome	*bla*$_{OXA-488}$ (100/99.87)<br>*bla*$_{PAO}$ (100/99.24)<br>*bla*$_{OXA-35}$ (100/100)	*bla*$_{VIM-4}$ (100/100)
P.aeruginosa E6130952	NZ_CP020603<br>NZ_CP020602	Chromosome<br>plasmid(pJHX613)	*bla*$_{OXA-488}$ (100/99.87)<br>*bla*$_{PAO}$ (100/99.24)	*bla*$_{OXA-2}$ (100/100)
P.aeruginosa FDAARGOS_570	NZ_CP033835<br>NZ_CP033834	Chromosome<br>plasmid (unnamed)	*bla*$_{OXA-396/494}$ (100/99.62)<br>*bla*$_{PAO}$ (100/99.83)<br>*bla*$_{OXA-10}$ (100/100)	*bla*$_{VIM-6}$ (100/100)
P.aeruginosa FDAARGOS_571	NZ_CP033833	Chromosome	*bla*$_{OXA-488}$ (100/99.87)<br>*bla*$_{PAO}$ (100/99.24)	*bla*$_{OXA-2}$ (100/100)
P.aeruginosa IMP-13	NZ_CP034354<br>NZ_CP034355	Chromosome<br>plasmid (pPYO_TB)	*bla*$_{OXA-50}$ (100/99.61)<br>*bla*$_{PAO}$ (100/99.41)	*bla*$_{IMP-13}$ (100/100)
P.aeruginosa IOMTU 133	NZ_AP017302	Chromosome	*bla*$_{OXA-488}$ (100/100)<br>*bla*$_{PAO}$ (100/99.33)	*bla*$_{DIM-1}$ (100/100)

P.aeruginosa K34-7	NZ_CP029707	Chromosome plasmid (pK34-7-1)	*bla*$_{OXA-486}$ (100/99.74)<br>*bla*$_{PAO}$ (100/99.49)<br>*bla*$_{OXA-4}$ (100/100)	*bla*$_{VIM-2}$ (100/100)
P.aeruginosa N15_01092	NZ_CP012901	Chromosome	*bla*$_{OXA-396}$ (100/100)<br>*bla*$_{PAO}$ (100/99.66)	*bla*$_{NDM-1}$ (100/100)
P.aeruginosa NCGM_1900	NZ_AP014622	Chromosome	*bla*$_{OXA-488}$ (100/99.87)<br>*bla*$_{PAO}$ (100/99.24)	*bla*$_{IMP-34}$ (100/100)
P.aeruginosa NCGM_1984	NZ_AP014646	Chromosome	*bla*$_{OXA-488}$ (100/99.87)<br>*bla*$_{PAO}$ (100/99.24)	*bla*$_{IMP-34}$ (100/100)
P.aeruginosa NCGM2_S1	NC_017549	Chromosome	*bla*$_{OXA-488}$ (100/99.87)<br>*bla*$_{PAO}$ (100/99.24)<br>bla$_{TEM-1b}$ (100/100)	*bla*$_{IMP-1}$ (100/100)
P.aeruginosa NCGM257	NZ_AP014651	Chromosome	*bla*$_{OXA-50}$ (100/99.36)<br>*bla*$_{PAO}$ (100/99.49)<br>*bla*$_{OXA-4}$ (100/100)	*bla*$_{IMP-1}$ (100/100)
P.aeruginosa NCTC13715	NZ_LR134330	Chromosome	*bla*$_{OXA-395}$ (100/99.87)<br>*bla*$_{PAO}$ (100/99.33)<br>*bla*$_{OXA-10}$ (100/100)	*bla*$_{VIM-2}$ (100/100)<br>*bla*$_{VIM-48}$ (100/99.86)
P.aeruginosa Pa58	NZ_CP021775	Chromosome	*bla*$_{OXA-488}$ (100/100)<br>*bla*$_{PAO}$ (100/99.24)	*bla*$_{GES-1}$ (100/100)<br>*bla*$_{OXA-2}$ (100/100)
P.aeruginosa PA83	NZ_CP017293<br>NZ_CP017294	Chromosome plasmid (unnamed)	*bla*$_{OXA-486}$ (100/99.74)<br>*bla*$_{PAO}$ (100/99.49)<br>*bla*$_{OXA-4}$ (100/100)	*bla*$_{VIM-2}$ (100/100)

P.aeruginosa Pa124	NZ_CP021774	Chromosome	*bla*$_{OXA-50}$ (100/99.23)<br>*bla*$_{PAO}$ (100/99.24)	*bla*$_{GES-19}$ (100/100)<br>*bla*$_{GES-20}$ (100/100)
P.aeruginosa Pa127	NZ_CP022000	Chromosome	*bla*$_{OXA-50}$ (100/99.23)<br>*bla*$_{PAO}$ (100/99.24)	*bla*$_{GES-19}$ (100/100)<br>*bla*$_{GES-20}$ (100/100)
P.aeruginosa PA298	NZ_CP040127<br>NZ_CP040126	Chromosome<br>plasmid<br>(pBM908)	*bla*$_{OXA-396/494}$ (100/99.62)<br>*bla*$_{PAO}$ (100/99.58)<br>*bla*$_{OXA-1}$ (100/99.87)	*bla*$_{VIM-1}$ (100/100)<br>*bla*$_{IMP-45}$ (100/100)
P.aeruginosa PA1088	NZ_CP015001	Chromosome	*bla*$_{OXA-396/494}$<br>(100/99.62)*bla*$_{PAO}$<br>(100/99.58)*bla*$_{OXA-56}$<br>(100/100)	*bla*$_{SPM-1}$ (100/100)
P.aeruginosa Pa1207	NZ_CP022001	Chromosome	*bla*$_{OXA-396}$ (100/100)<br>bla$_{PAO}$ (100/99.24)	*bla*$_{OXA-2}$ (100/100)
P.aeruginosa PA7790	NZ_CP014999<br>NZ_CP015000	Chromosome<br>plasmid<br>(pPA7790)	*bla*$_{OXA-396/494}$ (100/99.62)<br>*bla*$_{PAO}$ (100/99.58)<br>*bla*$_{OXA-56}$ (100/100)	*bla*$_{SPM-1}$ (100/100)
P.aeruginosa PA8281	NZ_CP015002	Chromosome	*bla*$_{OXA-396/494}$ (100/99.62)<br>*bla*$_{PAO}$ (100/99.58)<br>*bla*$_{OXA-56}$ (100/100)	*bla*$_{SPM-1}$ (100/100)<br>*bla*$_{SPM-1}$ (100/100)
P.aeruginosa PA11803	NZ_CP015003	Chromosome	*bla*$_{OXA-396/494}$ (100/99.62)<br>*bla*$_{PAO}$ (100/99.58)<br>*bla*$_{OXA-56}$ (100/100)	*bla*$_{SPM-1}$ (100/100)

P.aeruginosa PA121617	NZ_CP016214<br>NZ_CP016215	Chromosome<br>plasmid (pBM413)	*bla*$_{OXA-50}$ (100/99.87)<br>*bla*$_{PAO}$ (100/99.92)<br>*bla*$_{OXA-1}$ (100/100)	*bla*$_{IMP-45}$ (100/100)
P.aeruginosa PASGNDM345	NZ_CP020703	Chromosome	*bla*$_{OXA-488}$ (100/100)<br>*bla*$_{PAO}$ (100/99.24)	*bla*$_{NDM-1}$ (100/100)
P.aeruginosa PASGNDM699	NZ_CP020704	Chromosome	*bla*$_{OXA-488}$ (100/100)<br>*bla*$_{PAO}$ (100/99.24)	*bla*$_{NDM-1}$ (100/100)
P.aeruginosa R31	NZ_CP061850<br>NZ_CP061851	Chromosome<br>plasmid (pR31-KPC)	*bla*$_{OXA-486}$ (100/99.87)<br>*bla*$_{PAO}$ (100/99.58)	*bla*$_{KPC-2}$ (100/100)
P.aeruginosa RIVM_EMC2982	NZ_CP016955	Chromosome	*bla*$_{OXA-395}$ (100/100)<br>*bla*$_{PAO}$ (100/99.49)<br>*bla*$_{CARB-2}$ (100/100)	*bla*$_{VIM-2}$ (100/100)
P.aeruginosa SP4371	NZ_CP034369	Chromosome	*bla*$_{OXA-50}$ (100/99.36)<br>*bla*$_{PAO}$ (100/99.49)<br>*bla*$_{VEB-1}$ (100/100)<br>*bla*$_{OXA-10}$ (100/100)<br>*bla*$_{PME-1}$ (100/100)	*bla*$_{NDM-1}$ (100/100)<br>*bla*$_{NDM-1}$ (100/100)
P.aeruginosa SP4527	NZ_CP034409	Chromosome	*bla*$_{OXA-50}$ (100/99.36)<br>*bla*$_{PAO}$ (100/99.49)<br>*bla*$_{VEB-1}$ (100/100)<br>*bla*$_{OXA-10}$ （100/100）<br>*bla*$_{PME-1}$ （100/100）	*bla*$_{NDM-1}$ (100/100)<br>*bla*$_{NDM-1/11}$ (100/99.87)

P.aeruginosa SP4528	NZ_CP033439	Chromosome	*bla*$_{OXA-50}$ (100/99.36)<br>*bla*$_{PAO}$ (100/99.49)<br>*bla*$_{VEB-1}$ (100/100)<br>*bla*$_{OXA-10}$ (100/100)<br>*bla*$_{PME-1}$ (100/100)	*bla*$_{NDM-1}$ (100/100)<br>*bla*$_{NDM-1/11}$ (100/99.87)
P.aeruginosa VRFPA04	NZ_CP008739	Chromosome	*bla*$_{OXA-488}$ (100/100)<br>*bla*$_{PAO}$ (100/100)<br>*bla*$_{TEM-1b}$ (100/100)	*bla*$_{VIM-2}$ (100/100)
P.aeruginosa 1705-19119	NZ_CP041773<br>NZ_MN208061	Chromosome plasmid (p519119-DIM)	*bla*$_{OXA-486}$ (100/99.49)<br>*bla*$_{PAO}$ (100/99.58)<br>*bla*$_{OXA-4}$(100/100)	*bla*$_{DIM-1}$ (100/99.73)
P.aeruginosa A681	NZ_CP041771NZ_MF344570	Chromosomeplasmid (pA681-IMP)	*bla*$_{OXA-486}$ (100/99.87)*bla*$_{PAO}$ (100/99.33)*bla*$_{PER-1}$ (100/100)*bla*$_{OXA-1}$ (100/100)	*bla*$_{IMP-45}$ (100/100)
P.aeruginosa SP2230	NZ_CP034434	Chromosome	*bla*$_{OXA-50}$ (100/99.36)<br>*bla*$_{PAO}$ (100/99.49)<br>*bla*$_{PAO}$ (100/98.91)	Not detected
P.aeruginosa B17932	NZ_CP034436	Chromosome	*bla*$_{OXA-50}$ (100/99.36)<br>*bla*$_{PAO}$ (100/99.49)<br>*bla*$_{OXA-10/233}$ (100/99.87)<br>*bla*$_{VEB-1}$ (100/100)	Not detected
P.aeruginosa 12-4-4(59)	NZ_CP013696	Chromosome	*bla*$_{OXA-50}$(100/99.62)<br>*bla*$_{PAO}$(100/99.58)	Not detected

P.aeruginosa 268	NZ_CP032761	Chromosome	*bla*$_{OXA-488}$ (100/99.87)<br>*bla*$_{PAO}$ (100/99.24)	Not detected
P.aeruginosa 8380	NZ_AP014839	Chromosome	*bla*$_{OXA-486}$ (100/99.87)<br>*bla*$_{PAO}$ (100/99.58)	Not detected
P.aeruginosa 60503	NZ_CP041774	Chromosome	*bla*$_{OXA-395}$ (100/99.87)<br>*bla*$_{PAO}$ (100/99.33)	Not detected
P.aeruginosa 243931	NZ_CP041772	Chromosome	*bla*$_{OXA-488}$ (100/99.87)<br>*bla*$_{PAO}$ (100/99.24)	Not detected
P.aeruginosa AES1M	NZ_CP037925	Chromosome	*bla*$_{OXA-50/396/494}$ (100/99.74)<br>*bla*$_{PAO}$ (100/99.58)	Not detected
P.aeruginosa AES1R	NZ_CP037926	Chromosome	*bla*$_{OXA-50/396/494}$ (100/99.74)<br>*bla*$_{PAO}$ (100/99.58)	Not detected
P.aeruginosa AR_0095	NZ_CP027538	Chromosome	*bla*$_{OXA-395}$ (100/99.36)<br>*bla*$_{PAO}$ (100/99.33)	Not detected
P.aeruginosa AR_0354	NZ_CP027171	Chromosome	*bla*$_{OXA-488}$ (100/99.87)<br>*bla*$_{PAO}$ (100/99.24)	Not detected
P.aeruginosa AR_0357	NZ_CP027166	Chromosome	*bla*$_{OXA-488}$ (100/99.87)<br>*bla*$_{PAO}$ (100/99.24)<br>*bla*$_{OXA-10}$ (100/100)<br>*bla*$_{VEB-1}$ (100/100)	Not detected
P.aeruginosa AR_0360	NZ_CP027165	Chromosome	bla$_{OXA-486}$ (100/99.74)<br>bla$_{PAO}$ (100/99.49)	Not detected
P.aeruginosa AR_0446	NZ_CP029660	Chromosome	*bla*$_{OXA-50}$ (100/98.85)<br>*bla*$_{PAO}$ (100/97.73)	Not detected

P.aeruginosa AR_455	NZ_CP030328	Chromosome	*bla*$_{OXA-395}$ (100/99.61)<br>*bla*$_{PAO}$ (100/99.24)	Not detected
P.aeruginosa AR_458	NZ_CP030327	Chromosome	bla$_{OXA-486}$ (100/99.87)<br>bla$_{PAO}$ (100/99.24)	Not detected
P.aeruginosa AR_460	NZ_CP030351	Chromosome	bla$_{OXA-486}$ (100/99.74)<br>bla$_{PAO}$ (100/99.24)	Not detected
P.aeruginosa AR442	NZ_CP029090	Chromosome	*bla*$_{OXA-488}$ (100/99.49)<br>*bla*$_{PAO}$ (100/99.58)	Not detected
P.aeruginosa AR445	NZ_CP029088	Chromosome	*bla*$_{OXA-395}$ (100/100)*bla*$_{OXA-9}$ (100/100)*bla*$_{CARB-2}$ (100/100)*bla*$_{PAO}$ (100/99.49)	Not detected
P.aeruginosa ATCC 15692	NZ_CP017149	Chromosome	*bla*$_{OXA-50}$ (100/99.87)<br>*bla*$_{PAO}$ (100/100)	Not detected
P.aeruginosa ATCC 27853-1	NZ_CP015117	Chromosome	*bla*$_{OXA-396}$ (100/100)<br>*bla*$_{PAO}$ (100/99.24)	Not detected
P.aeruginosa ATCC 27853-2	NZ_CP011857	Chromosome	*bla*$_{OXA-396}$ (100/100)<br>*bla*$_{PAO}$ (100/99.24)	Not detected
P.aeruginosa B10W	NZ_CP017969	Chromosome	*bla*$_{OXA-488}$ (100/100)<br>*bla*$_{PAO}$ (100/99.24)	Not detected
P.aeruginosa B136-33	NC_020912	Chromosome	*bla*$_{OXA-488}$ (100/99.87)<br>*bla*$_{PAO}$ (100/99.24)	Not detected

P.aeruginosa B14130	NZ_CP034435	Chromosome	*bla*$_{OXA-50}$ (100/99.36)<br>*bla*$_{PAO}$ (100/99.49)<br>*bla*$_{OXA-10}$ (100/100)<br>*bla*$_{VEB-1}$ (100/100)	Not detected
P.aeruginosa BAMCPA07-48	NZ_CP015377	Chromosome	*bla*$_{OXA-488}$ (100/99.87)<br>*bla*$_{PAO}$ (100/98.99)	Not detected
P.aeruginosa CCUG 70744	NZ_CP023255	Chromosome	*bla*$_{OXA-488}$ (100/99.49)<br>*bla*$_{PAO}$ (100/99.58)	Not detected
P.aeruginosa Cu1510	NZ_CP013144	Chromosome	*bla*$_{OXA-488}$ (100/100)<br>*bla*$_{PAO}$ (100/99.49)	Not detected
P.aeruginosa DHS01	NZ_CP013993	Chromosome	*bla*$_{OXA-488}$ (100/99.49)<br>*bla*$_{PAO}$ (100/99.58)	Not detected
P.aeruginosa DK1 substr.NH57388A	NZ_LN870292	Chromosome	*bla*$_{OXA-396/494}$ (100/99.61)<br>*bla*$_{PAO}$ (100/99.58)	Not detected
P.aeruginosa DK2	NC_018080	Chromosome	*bla*$_{OXA-396/494}$ (100/99.61)<br>*bla*$_{PAO}$ (100/99.33)	Not detected
P.aeruginosa DN1	NZ_CP017099	Chromosome	*bla*$_{OXA-395}$ (100/100)<br>*bla*$_{PAO}$ (100/98.9)	Not detected
P.aeruginosa DSM 50071	NZ_CP012001	Chromosome	*bla*$_{OXA-50/396/494}$ (100/99.74)<br>*bla*$_{PAO}$ (100/99.58)	Not detected
P.aeruginosa E80	NZ_CP031677	Chromosome	*bla*$_{OXA-396/494}$ (100/99.61)<br>*bla*$_{PAO}$ (100/99.58)	Not detected
P.aeruginosa E90	NZ_CP044006	Chromosome	*bla*$_{OXA-50}$ (100/99.87)<br>*bla*$_{PAO}$ (100/99.41)	Not detected

P.aeruginosa early isolate NN2(clone C)	NZ_LT883143	Chromosome	*bla*$_{OXA-50}$ (100/100)<br>*bla*$_{PAO}$ (100/99.66)	Not detected
P.aeruginosa F5677	NZ_CP026680	Chromosome	*bla*$_{OXA-395}$ (100/100)<br>*bla*$_{PAO}$ (100/99.83)	Not detected
P.aeruginosa F9670	NZ_CP008873	Chromosome	*bla*$_{OXA-396}$ (100/100)<br>*bla*$_{PAO}$ (100/99.24)	Not detected
P.aeruginosa F9676	NZ_CP012066	Chromosome	*bla*$_{OXA-50/494}$ (100/99.74)<br>*bla*$_{PAO}$ (100/99.58)	Not detected
P.aeruginosa F22031	NZ_CP007399	Chromosome	*bla*$_{OXA-50}$ (100/99.87)<br>*bla*$_{PAO}$ (100/99.83)	Not detected
P.aeruginosa F23197	NZ_CP008856	Chromosome	bla$_{OXA-486}$ (100/99.61)bla$_{PAO}$ (100/99.66)	Not detected
P.aeruginosa F30658	NZ_CP008857	Chromosome	*bla*$_{OXA-395}$ (100/100)<br>*bla*$_{PAO}$ (100/99.49)	Not detected
P.aeruginosa F63912	NZ_CP008858	Chromosome	*bla*$_{OXA-50}$ (100/100)<br>*bla*$_{PAO}$ (100/99.74)	Not detected
P.aeruginosa FA-HZ1	NZ_CP017353	Chromosome	*bla*$_{OXA-396/494}$ (100/99.74)<br>*bla*$_{PAO}$ (100/99.49)	Not detected
P.aeruginosa FDAARGOS_501	NZ_CP033843	Chromosome	*bla*$_{OXA-488}$ (100/99.87)<br>*bla*$_{PAO}$ (100/99.24)	Not detected
P.aeruginosa FDAARGOS_505	NZ_CP033832	Chromosome	*bla*$_{OXA-395}$ (100/100)<br>*bla*$_{PAO}$ (100/99.58)	Not detected
P.aeruginosa FDAARGOS_532	NZ_CP033771	Chromosome	*bla*$_{OXA-396}$ (100/100)<br>*bla*$_{PAO}$ (100/99.58)	Not detected

P.aeruginosa FDAARGOS_610	NZ_CP041013	Chromosome	bla$_{OXA-486}$ (100/99.74)<br>bla$_{PAO}$ (100/99.66)	Not detected
P.aeruginosa FDAARGOS_767	NZ_CP041008	Chromosome	*bla*$_{OXA-50}$ (100/99.87)<br>*bla*$_{PAO}$ (100/100)	Not detected
P.aeruginosa FRD1	NZ_CP010555	Chromosome	*bla*$_{OXA-395}$ (100/100)<br>*bla*$_{PAO}$ (100/99.49)	Not detected
P.aeruginosa GIMC5015:PAKB6	NZ_CP034429	Chromosome	*bla*$_{OXA-50}$ (100/99.87)<br>*bla*$_{PAO}$ (100/100)	Not detected
P.aeruginosa H5708	NZ_CP008859	Chromosome	*bla*$_{OXA-486}$ (100/99.74)<br>*bla*$_{PAO}$ (100/99.74)	Not detected
P.aeruginosa H25883	NZ_CP033686	Chromosome	*bla*$_{OXA-395}$ (100/99.74)<br>*bla*$_{PAO}$ (100/99.33)	Not detected
P.aeruginosa H26023	NZ_CP033685	Chromosome	*bla*$_{OXA-488}$ (100/100)<br>*bla*$_{PAO}$ (100/99.33)	Not detected
P.aeruginosa H26027	NZ_CP033684	Chromosome	*bla*$_{OXA-50}$ (100/100)<br>*bla*$_{PAO}$ (100/99.66)	Not detected
P.aeruginosa H27930	NZ_CP008860	Chromosome	*bla*$_{OXA-50}$ (100/99.74)<br>*bla*$_{PAO}$ (100/99.91)	Not detected
P.aeruginosa H47921	NZ_CP008861	Chromosome	*bla*$_{OXA-395}$ (100/99.49)<br>*bla*$_{PAO}$ (100/99.58)	Not detected
P.aeruginosa HOU1	NZ_CP042269<br>NZ_CP042268	Chromosome<br>plasmid<br>（pHOU1-1）	*bla*$_{OXA-396/494}$ (100/99.74)<br>*bla*$_{PAO}$ (100/99.41)	Not detected

P.aeruginosa HS9	NZ_CP030861	Chromosome	*bla*$_{OXA-396/494}$ (100/99.74)<br>*bla*$_{PAO}$ (100/99.49)	Not detected
P.aeruginosa IMP66	NZ_CP028959	Chromosome	*bla*$_{OXA-396/494}$ (100/99.74)<br>*bla*$_{PAO}$ (100/99.66)	Not detected
P.aeruginosa IMP67	NZ_CP028848	Chromosome	*bla*$_{OXA-396/494}$ (100/99.74)<br>*bla*$_{PAO}$ (100/99.66)	Not detected
P.aeruginosa IMP68	NZ_CP028849	Chromosome	*bla*$_{OXA-396/494}$ (100/99.74)<br>*bla*$_{PAO}$ (100/99.66)	Not detected
P.aeruginosa JB2	NZ_CP028917	Chromosome	*bla*$_{OXA-488}$ (100/99.74)<br>*bla*$_{PAO}$ (100/99.66)	Not detected
P.aeruginosa L10	NZ_CP019338	Chromosome	*bla*$_{OXA-488}$ (100/100)<br>*bla*$_{PAO}$ (100/99.33)	Not detected
P.aeruginosa LES431	NC_023066	Chromosome	*bla*$_{OXA-50}$ (100/99.87)*bla*$_{PAO}$ (100/99.74)	Not detected
P.aeruginosa LESB58	NC_011770	Chromosome	*bla*$_{OXA-50}$ (100/99.87)<br>*bla*$_{PAO}$ (100/99.74)	Not detected
P.aeruginosa LW	NZ_CP022478	Chromosome	*bla*$_{OXA-395}$ (100/99.87)<br>*bla*$_{PAO}$ (100/99.07)	Not detected
P.aeruginosa M18	NC_017548	Chromosome	*bla*$_{OXA-488}$ (100/99.49)<br>*bla*$_{PAO}$ (100/99.58)	Not detected
P.aeruginosa M1608	NZ_CP008862	Chromosome	*bla*$_{OXA-395}$ (100/99.87)<br>*bla*$_{PAO}$ (100/99.49)	Not detected
P.aeruginosa M37351	NZ_CP008863	Chromosome	*bla*$_{OXA-488}$ (100/100)<br>*bla*$_{PAO}$ (100/99.33)	Not detected

P.aeruginosa MRSN12280	NZ_CP028162	Chromosome	*bla*OXA-488 (100/99.87)<br>*bla*PAO (100/99.24)	Not detected
P.aeruginosa MTB-1	NC_023019	Chromosome	*bla*OXA-488 (100/100)<br>*bla*PAO (100/99.24)	Not detected
P.aeruginosa N17_1	NZ_CP014948	Chromosome	*bla*OXA-396/494 (100/99.74)<br>*bla*PAO (100/99.58)	Not detected
P.aeruginosa NCTC9433	LS483497	Chromosome	*bla*OXA-395 (100/99.11)<br>*bla*PAO (100/99.16)	Not detected
P.aeruginosa NCTC10332	NZ_LN831024	Chromosome	*bla*OXA-50/396/494 (100/99.74)<br>*bla*PAO (100/99.58)	Not detected
P.aeruginosa NCTC10728	NZ_LR134342	Chromosome	*bla*OXA-396/494 (100/99.49)<br>*bla*PAO (100/100)	Not detected
P.aeruginosa NCTC11445	NZ_LR134308	Chromosome	*bla*OXA-486 (100/99.87)<br>*bla*PAO (100/99.83)	Not detected
P.aeruginosa NCTC12903	NZ_LR134309	Chromosome	*bla*OXA-395 (100/100)<br>*bla*PAO (100/99.24)	Not detected
P.aeruginosa NCTC13359	NZ_LR590473	Chromosome	*bla*OXA-486 (100/99.74)<br>*bla*PAO (100/99.58)	Not detected
P.aeruginosa NCTC13618	NZ_LR590474	Chromosome	*bla*OXA-396/494 (100/99.74)<br>*bla*PAO (100/99.49)	Not detected
P.aeruginosa NCTC13620	NZ_LR590472	Chromosome	*bla*OXA-50 (100/100)<br>*bla*PAO (100/99.66)	Not detected
P.aeruginosa NHmuc	NZ_CP013479	Chromosome	*bla*OXA-396/494 (100/99.61)<br>*bla*PAO (100/99.58)	Not detected

P.aeruginosa Ocean_1155	NZ_CP022526	Chromosome	*bla*OXA-395 (100/100)<br>*bla*PAO (100/98.99)	Not detected
P.aeruginosa Ocean_1175	NZ_CP022525	Chromosome	*bla*OXA-395 (100/100)<br>*bla*PAO (100/98.99)	Not detected
P.aeruginosa PA_3	CP033084	Chromosome	*bla*OXA-396/494 (100/99.74)<br>*bla*PAO (100/99.58)	Not detected
P.aeruginosa PA_150577	NZ_CP017306	Chromosome	*bla*OXA-488 (100/99.49)<br>*bla*PAO (100/99.58)	Not detected
P.aeruginosa PA_154197	NZ_CP014866	Chromosome	*bla*OXA-396 (100/100)<br>*bla*PAO (100/99.66)	Not detected
P.aeruginosa PA_D1	NZ_CP012585	Chromosome	*bla*OXA-395 (100/99.87)<br>*bla*PAO (100/99.49)	Not detected
P.aeruginosa PA_D2	NZ_CP012578	Chromosome	*bla*OXA-395 (100/99.87)<br>*bla*PAO (100/99.49)	Not detected
P.aeruginosa PA_D5	NZ_CP012579	Chromosome	*bla*OXA-395 (100/99.87)*bla*PAO (100/99.49)	Not detected
P.aeruginosa PA_D9	NZ_CP012580	Chromosome	*bla*OXA-395 (100/99.87)<br>*bla*PAO (100/99.49)	Not detected
P.aeruginosa PA_D16	NZ_CP012581	Chromosome	*bla*OXA-395 (100/99.87)<br>*bla*PAO (100/99.49)	Not detected
P.aeruginosa PA_D21	NZ_CP012582	Chromosome	*bla*OXA-395 (100/99.87)<br>*bla*PAO (100/99.49)	Not detected
P.aeruginosa PA_D22	NZ_CP012583	Chromosome	*bla*OXA-395 (100/99.87)<br>*bla*PAO (100/99.49)	Not detected

P.aeruginosa PA_D25	NZ_CP012584	Chromosome	*bla*$_{OXA-395}$ (100/99.87)<br>*bla*$_{PAO}$ (100/99.49)	Not detected
P.aeruginosa PA1	NC_022808	Chromosome	*bla*$_{OXA-50}$ (100/99.61)<br>*bla*$_{PAO}$ (100/99.49)	Not detected
P.aeruginosa PA1R	NC_022806	Chromosome	*bla*$_{OXA-50}$ (100/99.61)<br>*bla*$_{PAO}$ (100/99.49)	Not detected
P.aeruginosa PA1RG	NZ_CP012679	Chromosome	*bla*$_{OXA-50}$ (100/99.61)<br>*bla*$_{PAO}$ (100/99.49)	Not detected
P.aeruginosa PA14Or_reads	NZ_LT608330	Chromosome	*bla*$_{OXA-488}$ (100/100)<br>*bla*$_{PAO}$ (100/99.33)	Not detected
P.aeruginosa PA34	NZ_CP032552	Chromosome	*bla*$_{OXA-488}$ (100/99.87)<br>*bla*$_{PAO}$ (100/99.24)	Not detected
P.aeruginosa Pa84	NZ_CP021999	Chromosome	*bla*$_{OXA-50}$ (100/99.74)<br>*bla*$_{PAO}$ (100/99.74)	Not detected
P.aeruginosa Pa1242	NZ_CP022002	Chromosome	*bla*$_{OXA-396/494}$ (100/99.61)<br>*bla*$_{PAO}$ (100/99.58)	Not detected
P.aeruginosa PABL012	NZ_CP031659	Chromosome	*bla*$_{OXA-50}$ (100/99.87)<br>*bla*$_{PAO}$ (100/99.58)	Not detected
P.aeruginosa PABL017	NZ_CP031660	Chromosome	*bla*$_{OXA-50}$ (100/99.74)<br>*bla*$_{PAO}$ (100/99.66)	Not detected
P.aeruginosa PABL048	NZ_CP039293<br>NZ_CP039294	Chromosome<br>plasmid<br>(pPABL048)	*bla*$_{OXA-395}$ (100/99.61)<br>*bla*$_{PAO}$ (100/99.24)<br>*bla*$_{OXA-10}$ (100/100)	Not detected

P.aeruginosa PAER4_119	NZ_CP013113	Chromosome	*bla*$_{OXA-50/396/494}$ (100/99.74)<br>*bla*$_{PAO}$ (100/99.58)	Not detected
P.aeruginosa paerg000	NZ_LR130528	Chromosome	*bla*$_{OXA-396/494}$ (100/99.74)<br>*bla*$_{PAO}$ (100/99.41)	Not detected
P.aeruginosa paerg002	NZ_LR130527	Chromosome	*bla*$_{OXA-488}$ (100/99.49)<br>*bla*$_{PAO}$ (100/99.58)	Not detected
P.aeruginosa paerg003	NZ_LR130530	Chromosome	*bla*$_{OXA-488}$ (100/99.49)<br>*bla*$_{PAO}$ (100/99.58)	Not detected
P.aeruginosa paerg004	NZ_LR130531	Chromosome	*bla*$_{OXA-488}$ (100/99.49)<br>*bla*$_{PAO}$ (100/99.58)	Not detected
P.aeruginosa paerg005	NZ_LR130534	Chromosome	*bla*$_{OXA-50}$ (100/99.23)<br>*bla*$_{PAO}$ (100/99.24)	Not detected
P.aeruginosa paerg009	NZ_LR130533	Chromosome	*bla*$_{OXA-50}$ (100/99.23)<br>*bla*$_{PAO}$ (100/99.24)	Not detected
P.aeruginosa paerg010	NZ_LR130536	Chromosome	*bla*$_{OXA-488}$ (100/99.49)*bla*$_{PAO}$ (100/99.58)	Not detected
P.aeruginosa paerg011	NZ_LR130535	Chromosome	*bla*$_{OXA-488}$ (100/99.49)<br>*bla*$_{PAO}$ (100/99.58)	Not detected
P.aeruginosa paerg012	NZ_LR130537	Chromosome	*bla*$_{OXA-488}$ (100/99.49)<br>*bla*$_{PAO}$ (100/99.58)	Not detected
P.aeruginosa PAK_1	LR657304	Chromosome	*bla*$_{OXA-396/494}$ (100/99.74)<br>*bla*$_{PAO}$ (100/99.16)	Not detected
P.aeruginosa PAK_2	NZ_CP020659	Chromosome	*bla*$_{OXA-396/494}$ (100/99.74)<br>*bla*$_{PAO}$ (100/99.16)	Not detected

P.aeruginosa PAO1	NC_002516	Chromosome	*bla*$_{OXA-50}$ (100/99.87)<br>*bla*$_{PAO}$ (100/100)	Not detected
P.aeruginosa PAO1_Orsay	NZ_LN871187	Chromosome	*bla*$_{OXA-50}$ (100/99.87)<br>*bla*$_{PAO}$ (100/100)	Not detected
P.aeruginosa PAO1161	NZ_CP032126	Chromosome	*bla*$_{OXA-50}$ (100/99.87)<br>*bla*$_{PAO}$ (100/100)	Not detected
P.aeruginosa PB350	NZ_CP025055	Chromosome	*bla*$_{OXA-488}$ (100/99.87)<br>*bla*$_{PAO}$ (100/99.16)	Not detected
P.aeruginosa PB353	NZ_CP025051<br>NZ_CP025052	Chromosome<br>plasmid<br>(pPB353_1)	*bla*$_{OXA-50}$ (100/99.61)<br>*bla*$_{PAO}$ (100/99.83)<br>*bla*$_{CTX-M-30}$ (100/99.77)<br>*bla*$_{OXA-101}$ (100/100)<br>*bla*$_{TEM-1B}$ (100/100)	Not detected
P.aeruginosa PB354	NZ_CP025053	Chromosome	*bla*$_{OXA-50}$ (100/99.61)<br>*bla*$_{PAO}$ (100/99.83)<br>*bla*$_{CTX-M-30}$ (100/99.77)<br>*bla*$_{OXA-101}$ (100/100)<br>*bla*$_{TEM-1B}$ (100/100)	Not detected
P.aeruginosa PB367	NZ_CP025056	Chromosome	*bla*$_{OXA-488}$ (100/99.87)<br>*bla*$_{PAO}$ (100/99.24)	Not detected
P.aeruginosa PB368	NZ_CP025050	Chromosome	*bla*$_{OXA-488}$ (100/99.87)<br>*bla*$_{PAO}$ (100/99.07)	Not detected
P.aeruginosa PB369	NZ_CP025049	Chromosome	*bla*$_{OXA-488}$ (100/99.87)<br>*bla*$_{PAO}$ (100/98.99)	Not detected

P.aeruginosa PcyII-10	NZ_LT673656	Chromosome	*bla*$_{OXA-485/488}$ (100/99.36)<br>*bla*$_{PAO}$ (100/99.58)	Not detected
P.aeruginosa PPF-1	NZ_CP023316	Chromosome	*bla*$_{OXA-395}$ (100/99.23)<br>*bla*$_{PAO}$ (100/99.99)	Not detected
P.aeruginosa RP73	NC_021577	Chromosome	*bla*$_{OXA-50}$ (100/100)<br>*bla*$_{PAO}$ (100/99.74)	Not detected
P.aeruginosa RW109	NZ_LT969520<br>NZ_LT969519<br>NZ_LT969521	Chromosome<br>plasmid1<br>(unnamed)<br>plasmid2<br>(unnamed)	*bla*$_{OXA-395}$ (100/100)<br>*bla*$_{PAO}$ (100/99.83)	Not detected
P.aeruginosa S86968	NZ_CP008865	Chromosome	*bla*$_{OXA-396}$ (100/100)<br>*bla*$_{PAO}$ (100/99.24)	Not detected
P.aeruginosa SCV20265	NC_023149	Chromosome	*bla*$_{OXA-50/396/494}$ (100/99.74)<br>*bla*$_{PAO}$ (100/99.74)	Not detected
P.aeruginosa SCVFeb	NZ_CP013477	Chromosome	*bla*$_{OXA-396/494}$ (100/99.61)<br>*bla*$_{PAO}$ (100/99.58)	Not detected
P.aeruginosa SCVJan	NZ_CP013478	Chromosome	*bla*$_{OXA-396/494}$ (100/99.61)<br>*bla*$_{PAO}$ (100/99.58)	Not detected
P.aeruginosa SJTD-1	NZ_CP015877	Chromosome	*bla*$_{OXA-486}$ (100/99.87)<br>*bla*$_{PAO}$ (100/99.58)	Not detected
P.aeruginosa T38079	NZ_CP008866	Chromosome	*bla*$_{OXA-396}$ (100/100)<br>*bla*$_{PAO}$ (100/99.24)	Not detected
P.aeruginosa T52373	NZ_CP008867	Chromosome	*bla*$_{OXA-50/396/494}$ (100/99.74)<br>*bla*$_{PAO}$ (100/99.16)	Not detected

P.aeruginosa T63266	NZ_CP008868	Chromosome	*bla*$_{OXA-396/494}$ (100/99.74)<br>*bla*$_{PAO}$ (100/99.66)	Not detected
P.aeruginosa UCBPP-PA14	NC_008463	Chromosome	*bla*$_{OXA-488}$ (100/100)<br>*bla*$_{PAO}$ (100/99.33)	Not detected
P.aeruginosa USDA-ARS-USMARC-41639	NZ_CP013989	Chromosome	*bla*$_{OXA-396/494}$ (100/99.87)<br>*bla*$_{PAO}$ (100/99.66)	Not detected
P.aeruginosa VA-134	NZ_CP013245	Chromosome	*bla*$_{OXA-486}$ (100/99.87)<br>*bla*$_{PAO}$ (100/99.74)	Not detected
P.aeruginosa W16407	NZ_CP008869	Chromosome	*bla*$_{OXA-396/494}$ (100/99.61)<br>*bla*$_{PAO}$ (100/99.83)	Not detected
P.aeruginosa W36662	NZ_CP008870	Chromosome	*bla*$_{OXA-50}$ (100/100)<br>*bla*$_{PAO}$ (100/99.66)	Not detected
P.aeruginosa W45909	NZ_CP008871	Chromosome	*bla*$_{OXA-396/494}$ (100/99.74)<br>*bla*$_{PAO}$ (100/99.49)	Not detected
P.aeruginosa W60856	NZ_CP008864	Chromosome	*bla*$_{OXA-50}$ (100/99.87)<br>*bla*$_{PAO}$ (100/99.41)	Not detected
P.aeruginosa WCHPA075019	NZ_CP028584	Chromosome	*bla*$_{OXA-396/494}$ (100/99.61)<br>*bla*$_{PAO}$ (100/99.58)	Not detected
P.aeruginosa X78812	NZ_CP008872	Chromosome	*bla*$_{OXA-395}$(100/99.87)<br>*bla*$_{PAO}$ (100/99.58)	Not detected
P.aeruginosa Y31	NZ_CP030910	Chromosome	*bla*$_{OXA-486}$ (100/99.61)<br>*bla*$_{PAO}$ (100/99.58)	Not detected

P.aeruginosa Y71	NZ_CP030911	Chromosome	*bla*$_{OXA-396/496}$ (100/99.61)<br>*bla*$_{PAO}$ (100/99.58)<br>*bla*$_{OXA-1}$ (100/100)	Not detected
P.aeruginosa Y82	NZ_CP030912	Chromosome	*bla*$_{OXA-395}$ (100/100)<br>*bla*$_{PAO}$ (100/99.49)<br>*bla*$_{OXA-1}$ (100/100)	Not detected
P.aeruginosa Y89	NZ_CP030913<br>NZ_CP030914	Chromosome<br>plasmid<br>（pY89）	*bla*$_{OXA-396/496}$ (100/99.61)<br>*bla*$_{PAO}$ (100/99.58)<br>*bla*$_{OXA-1}$ (100/100)	Not detected
P.aeruginosa YL84	NZ_CP007147	Chromosome	*bla*$_{OXA-396}$ (100/100)<br>*bla*$_{PAO}$ (100/99.33)	Not detected
P.aeruginosa AR_0356	NZ_CP027169<br>NZ_CP027168<br>NZ_CP027170<br>NZ_CP027167	Chromosome<br>plasmid<br>(unnamed)<br>plasmid<br>(unnamed)<br>plasmid<br>(unnamed)	Not detected	*bla*$_{KPC-2}$
P.aeruginosa AR441	NZ_CP029093NZ_CP029091NZ_CP02909<br>2NZ_CP029094	Chromosomep<br>lasmid<br>(unnamed)plas<br>mid<br>(unnamed)plas<br>mid (unnamed)	Not detected	*bla*$_{KPC-2}$

P.aeruginosa AZPAE15042	NZ_CP041354<br>NZ_CP041355	Chromosome<br>plasmid<br>(pIHMA87)	Not detected	Not detected
P.aeruginosa PA7	NC_009656	Chromosome	Not detected	Not detected
P.aeruginosa NCTC13718	LS483402	Chromosome	Not detected	Not detected
P.aeruginosa CR1	NZ_CP020560	Chromosome	Not detected	Not detected
